# Computational Techniques for Eye Movements Analysis towards Supporting Early Diagnosis of Alzheimer's Disease: A Review

**DOI:** 10.1155/2018/2676409

**Published:** 2018-05-20

**Authors:** Jessica Beltrán, Mireya S. García-Vázquez, Jenny Benois-Pineau, Luis Miguel Gutierrez-Robledo, Jean-François Dartigues

**Affiliations:** ^1^Instituto Politécnico Nacional-CITEDI, Tijuana, BC, Mexico; ^2^CONACYT, Ciudad de México, Mexico; ^3^LaBRI, University of Bordeaux, Bordeaux, France; ^4^Instituto Nacional de Geriatría, Ciudad de México, Mexico; ^5^INSERM, University of Bordeaux, Bordeaux, France

## Abstract

An opportune early diagnosis of Alzheimer's disease (AD) would help to overcome symptoms and improve the quality of life for AD patients. Research studies have identified early manifestations of AD that occur years before the diagnosis. For instance, eye movements of people with AD in different tasks differ from eye movements of control subjects. In this review, we present a summary and evolution of research approaches that use eye tracking technology and computational analysis to measure and compare eye movements under different tasks and experiments. Furthermore, this review is targeted to the feasibility of pioneer work on developing computational tools and techniques to analyze eye movements under naturalistic scenarios. We describe the progress in technology that can enhance the analysis of eye movements everywhere while subjects perform their daily activities and give future research directions to develop tools to support early AD diagnosis through analysis of eye movements.

## 1. Introduction

Neurodegenerative diseases are a group of disorders characterized by the progressive degeneration of the neurons of the central or peripheral nervous systems. The degeneration affects neuron synapsis or produces neuron death [1]. The most frequent neurodegenerative diseases are Alzheimer's disease (AD) and Parkinson disease (PD) [2, 3]. According to the Alzheimer's Association (https://www.alz.org/), currently there are 5.7 million people living with AD only in the US and it is expected that this number would increase to 13.8 million by 2050 [4]. Although there is no cure for AD [5], several treatments have been tested [6], for example, currently approved drugs such as donepezil, galantamine, and rivastigmine [7] and nonpharmacologic therapies [4].

Alzheimer' disease is frequently diagnosed at late stages when symptoms have become evident, which occurs after a process of months or years of neuron degeneration [6]. However, when diagnosed at early stages, treatment helps to overcome the symptoms and improves the quality of life [2, 6] and offers to caregivers the opportunity to adapt and prepare the characteristics changes of dementia [8]. Also, the early diagnosis would allow testing the administration of more aggressive therapy to prevent AD development [9]. Despite many efforts, the noninvasive diagnosis of AD at early stages remains unsolved [5, 10].

Recent literature reviews have outlined robust findings demonstrating that eye movements abnormalities are sign of cognitive decline [11, 12] and can eventually be used to assess AD disease progression. Furthermore, current technology provides noninvasive equipment and methods to assess visual deficits ubiquitously and objectively in naturalistic scenarios [13]. An example is the use of eye trackers, which are devices that measure gaze fixation and saccadic motions of eyes. Eye trackers have been used in experiments of oculomotor performance related to AD diagnosis [14]. However, currently eye trackers have been used only in controlled laboratory settings. To analyze eye movements in naturalistic scenarios, such as in activities of daily living (ADL), besides the gaze fixation points, understanding the scene is required. The understanding of the scene can be achieved through analysis of recorded video with computer vision techniques. The computational analysis of the video, supported by the areas of psychology and neurology, allows distinguishing the items from the scenes that grab the attention of the viewer [15, 16]. This information can be used to compare the areas of interest from people with AD (PwAD) and control groups (people without AD) when observing natural scenes with a potential use in early detection.

In this paper, we firstly describe technological tools and methods that have been used to gather eye movements data. Then, we review existing research that has encountered relation between eye movements and AD. This section also describes a evolution of research on eye movements and AD since earliest research towards naturalistic scenarios more suitable for early detection.  Section 4 describes computational techniques that are useful for complementing the analysis of eye movements and AD in naturalistic scenarios. Finally, Section 5 includes the conclusion and future directions.

## 2. Data Collection

To collect data, an important step is choosing a proper eye tracker device according to a planned research study. Eye trackers are devices that measure the point of gaze or eye movements from an individual [17]. The availability of eye movements recordings allows researchers to gather and analyze ground truth data about visual exploration [18]. This feature makes eye trackers a useful tool to study changes in cognition through eye movements analysis [19, 20]. The cognitive process are not directly measured with eye trackers. We can manipulate independent variables according to experimental design setups and measure the behavioral response from participants with eye movements measures [21].

Eye tracking provides a noninvasive tool without contraindications suitable for potential screening and tracking of AD [22]. Eye trackers provide data sensitivity that makes them suitable for analyzing oculomotor abnormalities in AD. However, there are different technological approaches for the construction of eye trackers [21]. For this reason, it is important to choose properly the adequate eye tracker features that fit the study.

Eye trackers can be static or provide mobility. For example, there are screen-based eye trackers, as the one used in [23]. These types of eye trackers are desktop mounted and collect fixation points only from the gaze towards the content displayed on a screen. Another type of eye trackers is head mounted, as the “ExpressEye” used in [19]. In this case the device allows capturing gaze fixations not limited to a specific screen; however, the user can not freely move because the apparatus is cumbersome. Nowadays, there are available commercial mobile eye trackers, such as Tobbi© (https://www.tobiipro.com) or SMI© (https://www.smivision.com) that provide continuous, remote, and pervasive capture capabilities. These capabilities are desirable to analyze eye movements from PwAD in naturalistic scenarios.

Despite capabilities provided by eye trackers, there are concerns about the use of eye trackers when participants are unrestrained [24]. The concerns are about the reliability of gaze recording when capturing data when participants take a nonoptimal pose. This later might represent a challenge in naturalistic experimental setups.

To gather data from participants in AD studies, researches divide participants into groups. Usually, the groups reported in the literature are young controls, elder controls, and PwAD. However, there are studies that also include a group people with Mild Cognitive Decline (MCI) to differentiate between persons in a more advanced stage of AD. The cognitive status from participants is usually evaluated through neuropsychological tests, such as the Mini Mental State Examination (MMSE) [12]. However, some studies have used other techniques such as thyroid function test and magnetic resonance images [11] among others.

The participants perform instructed oculomotor tasks while observing visual stimuli, such as images or video. The fixation points from the participants are collected using the chosen eye tracking device. Then, statistical tests and other modeling techniques are applied for data analysis. Finally, results are presented correlating outcome measures with a cognitive status and by showing differences among control groups and PwAD if present. In the next section different approaches that have encountered relation between eye movements and AD under different experimental setups are described.

## 3. Eye Movements and Alzheimer's Disease

Several researches on predementia have reported manifestations of visual symptoms produced by senile plaques and tangles located in the visual regions of the brain [38, 39]. The pathological changes in the visual system caused by neurodegenerative diseases are reviewed in [40–43]. Examples of these pathological changes are visual acuity changes, atypical pupillary responses, and alteration in the oculomotor performance [44].

Eye movements involve a complex oculomotor control system formed by extensive cerebral regions [45]. Through post mortem studies, there is evidence that pathologies associated with AD affect the oculomotor brain regions [46, 47]. Altered eye movements patterns reflect the resulting underlying visuospatial and executive function impairments. Thus, movement patterns are related to higher cognitive control processes [48]. That is why, for example, eye movements allow exploring the cognitive process underlying visual search, providing information about how people forage and plan when performing visual search tasks [49].

Typically, the studies dedicated to explore relationships between eye movements and AD compare certain outcome measures between persons with AD (PwAD) and control groups when they accomplish a specific oculomotor tasks. Examples of these tasks are fixating on a given point [19] or watching a picture [12]. An example of a dependent variable on these studies is the reaction time to perform the given tasks.

To examine the research involving eye movements and Alzheimer's disease, we searched in the PubMed© (https://www.ncbi.nlm.nih.gov/pubmed) database with the keywords “Alzheimer's disease” and “Eye movements”. The database included 165 articles from 1979 to 2017. From the 165 articles, 14 are review articles and 80 articles are not related to AD; focus on atypical subsets of AD or eye movements are not part of the study. From the 165 results, we found that 71 articles are relevant to the research of oculomotor performance of AD patients. The 19.7% (14 articles) from the relevant articles were published in the last 3 years. This shows that research analyzing eye movements is gaining importance in AD studies. Furthermore, recent research shows that the analysis of oculomotor deficits is useful in early detection of AD and also has the potential to be used to assess disease progression [43, 50].

Table 1 shows a summary of the research indexed in PubMed since the last 5 years related to eye movements analysis and AD. The summary includes the references and years, the methods used by the researches, the main findings from the studies and information about the participants, and apparatus if present. In the following sections, we describe and categorize conducted research showing an evolution from early attempts towards more naturalistic scenarios.

### 3.1. Saccadic Eye Movements and Alzheimer's Disease

Traditional studies using saccadic eye movements (SEM) tasks have reported differences between PwAD and control groups. A saccade is a rapid motion of the eye (typically lasting between 30 to 80 ms to complete) [21]. Example of these studies includes prosaccades and antisaccades analysis [19, 51]. To study prosaccades, a participant has to saccade from a initial point to an appearing peripheral target. Then, the reaction time or latency is measured from the subject to fixate on the presented peripheral target. The research described in [37, 52, 53] reports increased latency of saccades from PwAD when compared to control groups that can be associated with cognitive process. On the other hand, to study antisaccades, the participant must fixate an opposite direction from a presented peripheral target [54]. As the participants have to inhibit the automatic saccade towards the stimulus, the antisaccade task requires additional executive processing from participants [55]. The nature of antisaccades can be associated with executive attention and research results indicate that patients with AD have shown more antisaccade errors with fewer corrections than control groups [56]. The papers [14, 44, 48, 50] review work conducted on eye movements and their relationship with AD.

There are different challenges regarding eye movements studies and AD. The first challenge arises because oculomotor abnormalities are not exclusive from AD and it is important to develop techniques that properly distinguish AD from other diseases. For example, SEM abnormalities have been encountered in Multiple System Atrophy, such as slower prosaccade and increased antisaccade errors [57].

Furthermore, SEM abnormalities might be related to aging. For example, in [58] it is reported that latencies uncorrected or increased time to correct error in antisaccades increase with aging. Older adults appear to have stronger difficulty ignoring distractions during day-to-day activities than younger adults. It seems that any variable that reduces the strength of the top-down neural signal to produce a voluntary saccade, or that increases saccade speed, will enhance the likelihood that a reflexive saccade to a stimulus with an abrupt onset will occur [59]. So, what is the effect of “normal” aging on eye saccade speed? It has been shown [60] that the Digit Symbol Substitution Test can be altered as far as 20 years before AD in older individuals with a high level of study is manifest. The performance in this test is related to the speed of eye saccades. This decline in performance speed and executive functions might be nonspecific prodromal Alzheimer's but could as well characterize a state of cerebral vulnerability on which the illness would progress more easily. Despite the relationship between age related cognitive decline and saccadic eye movements (SEM) deficits has been outlined, specific cognitive alterations underlying age-related changes in saccadic performance remain unclear. The nature of aging effects on SEMs has been only rarely approached. The progressive age-related decline of processing speed and executive attention is associated with and can be highlighted through saccadic age movement deficits as well in prosaccade and antisaccade tasks.

As can be see from Table 1, research from five years ago mainly focused in studying prosaccades and antisaccades. Indeed, the study of SEM was dominant since the earliest approaches dedicated to analyze eye movements and its relation to AD [52, 61–64]. As described, in SEM experiments the participants must fixate to a target. Although SEM studies have reported significant difference between persons with AD and control groups, there is still a research gap to fill in order to use SEM analysis as a marker for AD. Differentiate SEM abnormalities from AD, “normal” aging, and other conditions are among the main challenges from SEM analysis.

Prosaccade and antisaccade tasks have been popular in research studies due to their simplicity [58]. These tasks require a controlled scenario to conduct the evaluations. As research has evolved, more complex tasks have been studied towards associating eye movements deficits to support AD diagnosis. In Section 3.2 studies involving the execution of more complex tasks than attending to single target points are described. However, this research still lies in a category of controlled scenarios.

### 3.2. Eye Movements Analysis in Controlled Scenarios

Since the past years, research studies have moved forward to conduct other types of experiments aiming to identify eye movements abnormalities related to AD. For example, in [12] the participants performed a more complex task that only attend a target point that consisted in detecting and categorizing a specific object within a natural scene. The participants observed two visual stimuli in a monitor, one including an image with an animal and the other including a distractor image. The participants were asked to saccade to the image that contained the animal while their success to fixate to the animal and to the correct image was measured. The results from this study show that persons with AD, even in a mild stage of the disease, when compared with control groups have difficulties to select the relevant targets.

Another example is given by the work in [11] that focuses on the analysis of reading behavior of PwAD. Reading is an ADL that involves the use of working memory and memory retrieval function. Thus, the experiments in [11] involve analysis of more complex task than usual SEM studies. The experiment consisted in a comparison of the eye gaze position of PwAD and control participants when reading sentences. The findings from [11] show that PwAD have a longer gaze duration than controls. Additionally, they found that a predictability degree on the sentences is accounted by control subjects but not by PwAD. This suggests that PwAD have impairments with their working memory and memory retrieval functions. Although the work in [11] is towards analysis of eye movements in ADL, the current experiments are under controlled scenarios in the sense that use screen based eye trackers and even use a chin rest to constrain head movements. Another research studies the attention to repeated and novel stimuli [65] that is related to cognition and attention. The experiments consist in presenting slides to mild-to-moderate PwAD that contain novel and repeated images. The researchers report that fixations on the images serve to evaluate attendance to repeated and novel content providing the potential to be used to measure disease progression.

Another study analyzes the effects of AD on visual exploration [25]. The study focuses on visual search performance for target detection in the far periphery. The participants, AD patients, and control subjects explore a hemispherical screen and respond to presented targets. The results from this study show differences in AD patients and control subjects when identifying targets on different eccentricities from the screen. Researchers also report differences on target detection times and number of fixations. The work in [66] uses eye movements analysis during video watching to infer people's cognitive function. Researches defined 13 features from fixations and found correlations between the features and memory capability. Example of these features include mean fixation duration, fixation count, and mean saccade amplitude. Different from other specific laboratory tasks, in these experiments the participants freely watch videos from different scenarios while features from eye movements are extracted.

A pilot study in France, LYLO [67], focused on measuring the rigidity and lack of curiosity of PwAD. In this study, the patients were screened in laboratory settings with static images displayed. And statistical parameters computed from recordings of saccades and fixations were compared. A step forward measuring visual impairments in a naturalistic situation consists in using everyday visual content such as colour video content for patients screening. The lack of curiosity hence can be induced from gaze recordings of intentionally degraded natural video content. The baseline model of automatic prediction of attention for normal control subjects was developed in [67, 68]. Contrarily to [69] where the first step to make the observation “natural” was done by using video in free viewing conditions, in [68, 69] video was intentionally degraded.

While studying visual deficits on laboratory tasks has been productive for AD assessment, its application requires cooperative scenarios. That is, subjects must cooperate consciously performing the oculomotor tasks to get their assessment. Thus, this type of assessment is adequate when there is already an evident manifestation of dementia that requires evaluation. In addition, the manifestation cannot be severe enough so the subject is not able to cooperate. To achieve early diagnosis, it is necessary to have techniques that allow the evaluation of eye movements abnormalities on scenarios that allow naturalistic assessment without the explicit cooperation from subjects, for example, to analyze how AD affects eye movements in ADL, such as cooking or gardening. In the following section we describe the work related to analysis of eye movements in naturalistic scenarios.

### 3.3. Eye Movements Analysis in Naturalistic Tasks

When performing daily activities such as cooking or gardening, subjects interact with several objects, for example knifes, pans, or remote controls. During these interactions, a succession of different actions is involved, for example, cutting a vegetable or watering a plant. While executing these actions, humans use their vision to locate the objects and to manipulate them [70]. Indeed, the eye movements during everyday tasks provide relevant information about complex cognitive process related to object identification, place memory, tasks execution, and monitoring [71].

Studies attempt to understand the relation between activity execution and eye movements by investigating the eye patterns and eye-hand coordination on actions [72, 73]. For example, results support that people shift their gaze to target sites anticipating actions [74]. Also, results indicate that subjects rarely fixate on objects irrelevant to a performed action [73]. In fact, almost all eye movements during activities are targeted to fixation of task relevant objects, suggesting that visual attention can be modelled as “top-down” having little influence of the “intrinsic saliency” of the scene [72]. Top-down modelling refers to aspects of attention and gaze that are under executive control and may be influenced by tasks' directives and working memory [75]. Thus, top-down models require prior knowledge from the context [15]. On the other hand, bottom-up modelling refers to attention that is driven by properties of the visual stimulus being independent from tasks or semantic [75].

The described findings so far have arisen from experiments with healthy subjects. While the results are important to understand the eye movements in ADL, experiments with AD patients are scarce. This later is critical to support the diagnosis in early stages and to monitor changes of the disease. Healthy subjects have demonstrated different results when conducting specific oculomotor task when compared with PwAD and we expect the same in naturalistic scenarios.

In the work by Forde et al. [76] the eye movements in an ADL task are analyzed from a patient with action disorganization syndrome (ADS), from a PwAD, and from control subjects. The results show difference in the visual behavior from the participants while they were preparing a cup of tea. For example, the ADS patient made no glances to objects anticipating their use and had an increased number of fixations to irrelevant objects during the task. This shows different results from those stated in [73, 74]. The AD patient showed fewer fixations overall than control subjects and the ADS patients. In addition, the PwAD showed a lower proportion of relevant fixations compared to control subjects. In the work by Suzuki et al. [28] eye movement is investigated during locomotion. One AD patient, one Posterior Cortical Atrophy (PCA) patient, and one healthy subject used an eye tracking device while performing locomotion activities (walking along corridors, up and down stairs, and across a room with or without obstacles). The results show that the PCA patients were the slowest in performing the locomotion activities. Also, the PCA patient had longer fixation than the PwAD and the healthy subject. The PwAD required prompting during task competition showing memory impairment. Both studies show important findings toward understanding eye movements from PwAD; however more experiments with more participants are required.

A research goal is to understand eye movements abnormalities when performing ADL that serve to identify early signs of AD and to alert about a possible development of the disease. However, several challenges must be addressed first. For example, despite finding differences between PwAD and control groups, several abnormalities on visual deficits are not unique to AD but they are also present in other pathologies. In addition, it is important to find a visual marker that can be used to measure the progression of the disease; that is, the longitudinal studies must be conducted. Additionally, the clinical and personal history of each patient must be considered. PwAD might have differences in their visual behavior due to their physiological and personal context. Such is the case, for example, if they have sensorial impairments or a determining event in their lives. For example, a manual worker might have a different behavior than a white-collar worker.

The analysis of visual behavior during ADL has the potential to become a tool for AD assessment and for monitoring progression. Its success strongly relies on the development of technology able to measure eye movements. To be a pervasive tool it has to measure eye movements easily and in a noninvasive way allowing subjects to perform their activities in a natural manner.

Currently, there are clinical trials registers describing ongoing research with the objective to analyze eye movements in naturalistic tasks. For example, by doing a search with the terms “Alzheimer's disease” and “eye movements” in the database ClinicalTrials.gov provided by the US National Library of Medicine, there are 10 results currently recruiting participants. We identified 2 as relevant ongoing studies of eye movements in naturalistic scenarios for AD. In [77], researchers aim to analyze eye movements when reading sentences. In [78], researchers aim to analyze deficits in visual exploration in ADL.

Researchers in [78] expect to encounter that persons with AD have less ability of using scene semantic when locating objects. In this sense, the understanding of scene is paramount. In the next section we describe how the use of computational techniques can leverage the analysis of eye movements and AD by scene understanding.

## 4. Towards Early Detection Leveraging on Computational Attention Modelling

As we mentioned before, the identification of abnormalities in eye movements to support the early diagnosis or progression of an eventual dementia disease in elderly population during performance of ADL is a real scientific challenge. In this sense, some interesting approaches propose to predict human visual attention emulating the Human Visual Systems performance [79]. Indeed, computational visual attention models (CVSM) attempt to explain and describe the process of perceptual behavior and are compared with ground truth measured by eye trackers in psychovisual experiments [80–82]. Several exciting CVSM such as visual saliency techniques on egocentric video are useful for the use in naturalistic scenarios to estimate the areas from the video that are more likely to become the focus of human visual attention [15]. The egocentric video provides a first-person view from the individual who “wears” an egocentric camera giving visual information about objects, locations, and interactions.

Visual saliency techniques have been already combined with eye tracking in the field of Autism Spectrum Disorder (ASD) to screen differences between people with ASD and controls [83]. Researchers compare eye movements from both groups when freely viewing natural scenes images. In the analysis fixations towards visually salient regions, such as color, intensity, orientation, objects, and faces, are considered. To the best of our knowledge, research regarding the combination of data from eye trackers and visual saliency modeling to analyze eye movements abnormalities from PwAD is scarce. As we show earlier in Section 3.2 the work in [67] approaches with the analysis of fixations to degraded images, but more research is missing.

Suitable devices identified to be appropriated for monitoring ADLs with egocentric vision capabilities are mobile eye trackers [20], such as Tobbi© or SMI©. Additionally, egocentric cameras such as GoPro, Samsung Glass, and Microsoft Sense Cam [84] would allow scene understanding. Certainly, they record egocentric video giving a first-person view or in other words, what the camera wearer sees [85]. This captured information can be useful to analyze or predict some or all of visual attentive behavior through visual saliency computation. In this section, the main characteristics from the research on visual saliency are described, the techniques used and how this research field can be applied in the context of eye movements analysis. 

*(1) Computational Visual Saliency Models*. The research field that analyzes video computationally to estimate the image regions that attract visual attention is called visual saliency detection [86, 87]. This research field matches the areas of neuroscience, psychology, and computer vision [16].

Early work on visual saliency modelling uses handcrafted low-level features such as contrast [88], color [89], edges [90], and orientation. It is funded on feature integration theory by Treisman and Gelade [91]. In addition, there is work that performs higher-level features extraction such as objects [92] and faces [93] in order to incorporate, into the low-level features, semantic elements of the observed scenes. Moreover, since the boom of deep learning, there are different proposals of configuration and arrangements of these supervised classification tools such as the convolutional neural networks [94–96] that report increased results for saliency estimation tasks. However, as deep learning requires a huge amount of data, more annotated information on diverse scenarios is still required. The works in [15, 97, 98] review techniques used for visual saliency detection.

According to the method used for modeling attention, there are two main categories of methods followed in the research of visual saliency: bottom-up modelling and top-down modelling. Bottom-up methods use information such as color, contrast, orientation, and texture [99]; they predict stimuli driven attention. And top-down models require prior knowledge on the visual search task and the context [15]. Currently, most of the work in visual saliency enters in the category of bottom-up methods.

The visual saliency modelling outputs a saliency map S, which is a two-dimensional topographically arranged map that encodes stimulus conspicuity of the visual scene [100]. The pixel values in the map indicate the saliency degree of the corresponding regions in the visual scene [15]. The maps are compared against ground truth maps built upon gaze fixations recorded by eye trackers when subjects are performing visual tasks. The ground truth might include synthetic stimuli or come from natural scenes including still images and video [101].

Pioneer research on visual saliency studied saliency from a third-person perspective, for instance, using still images [102] or video [103] coming from nonegocentric cameras. In the scenario of understanding scene for AD studies, the interest is nevertheless in saliency from the point of view of the subject performing activities. Hence, building saliency maps in egocentric video content and from the point of view of the subjects wearing recording device is required. However, the analysis of egocentric video brings new challenges. For instance, motion cues are significant in third-person video but camera motion is inherent to egocentric recordings [104]. Therefore, a residual motion in image plane of egocentric video has to be computed after motion of camera wearer has been compensated [105]. Egocentric video allows better introducing contextual knowledge from the subject because egocentric video follows the field of view of the subject's action. In this sense, this paradigm can support top-down attention modeling [106] in naturalistic scenarios such as in ADLs execution.

Egocentric video, beside the bottom-up image cues, provides context about the manipulation of objects [107, 108], about hands positions [106, 109], ego-motion [110], actions [111, 112], and activities [113, 114]. The egocentric video supports top-down attention modelling by the ensemble of these diverse contextual information. For example, a model assumes that gaze goes towards a given object currently held by the subject's hands. In addition, the ego-motion that occurs in visual exploration towards locating a specific object that is required in a given activity also serves for gaze estimation [115].

Top-down and bottom-up computational visual attention modelling show correlation between human fixations and predicted saliency maps. However, most of the results arise from experiments including healthy participants while scarce studies involve PwAD. In this review, we focus on the scenario for early AD detection. Thus, we address the relevant work relating computational attention modelling applied to AD in the next section. 

*(2) Computational Visual Saliency Models and Diagnosis*. Progress in prediction of visual saliency including in egocentric content makes it possible to build robust prediction models for regions of high expectancy for the fixations of the test subject. Therefore, if a subject executing an ADL does not fixate the predicted areas properly, then it can be supposed that this subject has to undergo further tests to diagnose AD or not. For healthy subjects, visual saliency techniques assume that the subject will execute the activity fixating toward relevant objects or with coherent visual exploration. However, as mentioned in the work by Forde et al. [76], the participant with AD had lower proportion of relevant fixations compared to healthy subjects in ADL settings.

Another relevant feature about gaze measuring in patients with AD is the sensitivity. For example, the diagnosis performed with SEM tasks requires saccadic sensitivity. However, current visual saliency techniques addressing saccadic estimation are in early stages [116].

Although top-down mechanisms dominate the attention modelling from healthy subjects, it has been suggested that the visual behavior from persons with cognitive problems might rely on bottom-up mechanisms and saliency driven with less fixations on objects relevant to the task [71]. The work in [117] suggests that visual attention problems from AD patients are more notorious when the target item is not salient and shares common features with the background. The study in [118] analyzes the visual search task performance from AD patients by conducting experiments using salient and not salient search conditions. The researchers measure the reaction times when PwAD and control participants search for target elements. The PwAD show longer reaction times than control participants. However, the gap between both groups is bigger when searching for nonsalient target items. This suggests that salient elements attract PwAD.

The research in [105] on egocentric video acknowledges the potential to use visual saliency techniques to develop a tool for medical practitioners in realistic ADL scenarios. The researchers perform gaze comparison from an actor and a viewer. The actor is a person performing an activity (potentially a PwAD) while the viewer is a person watching egocentric video recordings from the actor (the medical practitioner). The paper suggests a relation between the gaze from the actor and the viewer. This relation consists in a time shift between the points of attention from the actor and the viewer. In other words, the viewer looks at the same place in the visual scene compared to the (healthy) actor but a few milliseconds later. The potential of this tool relies on the ability of a system to determine if the gaze from the actor is normal or abnormal according to the perspective from a medical practitioner. Additionally, the settings from the tool, like the use of egocentric camera, allow the use of computational attention-modelling techniques.

Computational attention-modelling techniques can be complemented with other contextual information in order to know more about the subject oculomotor behavior. For example, the amount of time that a participant takes to complete an activity can be explored. As literature shows, people with cognitive deficits take longer to complete tasks.

## 5. Conclusions and Future Directions

Neurodegenerative diseases, specifically Alzheimer's disease, are a problem that affects population worldwide. Currently, AD has no cure, but it has been demonstrated that treatment is helpful to delay the progression and improve quality of life. The diagnosis occurs frequently at late stages of the disease, when symptoms are evident. Nevertheless, research has found that AD is present up to 20 years before the disease is manifested. To have better treatment outcomes, it is desirable to have an early diagnosis. Understanding contextual differences that might influence the course of the disease would also be helpful. Among the current diagnostic techniques, visual behavior has the potential to become useful in early stages and to be a pervasive tool. Several investigations have explored the relations of eye movements with AD through specific oculomotor tasks demonstrating visual features that can be used for early diagnosis and progression measurement. However, more experiments are necessary under naturalistic scenarios to develop a useful tool that can be used in early stages. Nevertheless, changes happening in older individuals without cognitive impairment must also be taken into consideration and eventually they have to be approached by means of further research in normal individuals. Eye movements abnormalities have been measured mostly using eye tracking technology. Nevertheless, computer vision techniques, such as visual saliency and object detections in ADL performance settings, could be a good means to measure visual attention of PwAD to diagnose in terms of its difference to normal control attention in naturalistic scenarios when performing ADLs. Several challenges must be addressed, such as estimating gaze on top-down driven mechanisms and relating bottom-up mechanisms with the activities. Also, it is important to conduct experiments with persons with different cognitive problems in order to learn the features that differentiate among healthy subjects, people with different diseases, and persons with AD.

## Figures and Tables

**Table 1:** Research on eye movements and Alzheimer disease on pubmed since 2013.

Cite	Methods	Findings	Participants/Apparatus
2016 [25]	Subjects responded to targets presented on a hemispherical screen with diverse eccentricity.	PwAD recognized less targets in the center. No difference was found with CG on the peripheral targets.	AD: 18 CG: 20 Apparatus: Hemispherical screen Octupus 900 with camera used for eye tracking.

2017 [26]	The King-Devick test (with saccadic and other movements) was applied to subjects.	The King-Devick test may a tool to detect cognitive impairment associated with AD.	AD: 32 CG: 135 MCI: 39 Apparatus: N/A

2016 [27]	Subjects looked a series of slides containing four images of different emotional themes.	PwAD with apathy had diminished attentional bias toward social-themed stimuli.	AD: 36 (Apathy: 17 Not apathy: 19) Apparatus: Binocular eye tracking system developed by EL-MAR Inc.

2016 [11]	Eye movements from subjects were examined during reading regular and high predictable sentences.	PwAD gaze was longer than CG gaze. CG decreased gaze duration with high predictable sentences suggesting reading enhancement using stored information.	AD: 35 CG: 35 Apparatus: EyeLink 1000. Chinrest to control eye movements.

2015 [28]	Subjects performed a variety of tasks: walking, through stairs, through a room with and without obstacles.	The Posterior Cortical Atrophy (PCA) patient had longer mean fixation durations than PwAD and CG. Mean fixation duration between PwAD and CG was similar.	AD: 1 CG:1 PCA: 1 Apparatus: SMI mobile eye tracker

2015 [29]	Eye movements from subjects were examined while read sentences.	PwAD had more fixations on regular and high predictable sentences. PwAD spend more time reading the sentence. CG had less frequent second pass fixation over sentences.	AD: 35 CG-elderly: 35 Apparatus: EyeLink 1000. Chinrest to control eye movements.

2015 [19]	Longitudinal study with Gap and overlap paradigms.	PwAD had slower reaction times than CG. Prosaccades did not deteriorate after the 12-month longitudinal study in AD.	AD: 11 CG elderly: 25 Apparatus: ExpressEye

2015 [30]	Subjects made saccadic movement to photographs to target instructed scenes (natural vs urban, indoor vs outdoor)	Were found differences between controls and PwAD on accuracy but not saccadic latency.	AD: 24 CG age-matched: 28 CG young: 26 Apparatus: Eye tracker (Red-M, Senso-Motoric Instruments)

2015 [23]	Eye movements from subjects were examined while read proverbs.	PwAD have less word predictability than CG.	AD: 20 CG: 40 Apparatus: EyeLink 1000. Chinrest to control eye movements.

2014 [31]	Eye movements from subjects were examined while read low and high predictable sentences.	CG have shorter gaze duration on high predictable sentences. PwAD have similar gaze duration on both low and high predictable sentences. PwAD gaze duration is longer than CG.	AD: 20 CG age-matched: 40 Apparatus: EyeLink 1000. Chinrest to control eye movements.

2014 [32]	Eye movements from subjects were examined while read sentences	PwAD have altered visual exploration and absence on contextual predictability.	AD: 18 HC age-matched: 40 Apparatus: EyeLink 2K. Chinrest to control eye movements.

2013 [33]	Eye movements from subjects were examined while read sentences	PwAD evidences marked alterations in eye movement behavior during reading.	AD: 20 CG age-matched: 25 Apparatus: EyeLink 1000. Chinrest to control eye movements.

2014 [12]	Subjects were asked to spot an animal target contained in Colored photographs along with other distracting items.	PwAD were significate less accurate than elderly controls. Elder were less accurate than young controls.	AD: 17 mild AD. CG elderly: 23 CG young: 24. Apparatus: Eye tracker (Senso-Motoric Instruments)

2014 [34]	Subjects were required to look to a small fixation cross for 20 seconds on the center of a screen.	CG and PwAD showed significantly differences of microsaccade direction.	AD: 18 MCI: 15 CG age-matched: 21 Apparatus: Eye See Cam

2013 [35]	Visual targets were presented to subjects in a dim room. Prosaccade and antisaccade trials.	The antisaccade taks performance serves as a measure of executive function on PwAD.	AD: 28 MCI: 36 CG elderly: 118 Apparatus: Dual Purkinje Image Tracker. Heads stabilized on a chinrest.

2013 [36]	Pro-saccade and anti-saccade tasks. Gap and overlap paradigms.	PwAD have an excessive proportion of uncorrected errors in the antisaccade test.	AD: 18 Parkinson disease: 25 CG-young: 17 CG elderly: 18. Apparatus: Head mounted device ExpressEye eyetracker.

2013 [37]	Horizontal and vertical saccades. Gap and overlap paradigms on a black computer screen.	A link between MMSE and saccade latency.	AD: 25 Amnestic MCI: 18 CG elderly: 30 Apparatus: Head mounted Eyeseecam

CG: Control Group; MCI: Mild Cognitive Impairment; MMSE: Mini Mental State Examination.
